# Poly[(μ-2-hy­droxy-3,5-dinitro­benzoato)rubidium]

**DOI:** 10.1107/S1600536811008476

**Published:** 2011-03-15

**Authors:** Yan Meng

**Affiliations:** a School of Environmental Engineering, Chang’an University, South Second Cycle Road 368#, Xi’an 710064, Shaanxi, People’s Republic of China

## Abstract

The asymmetric unit of the title compound, [Rb(C_7_H_3_N_2_O_7_)]_*n*_, comprises an Rb^+^ cation and a 3,5-dinitro­salicylate ligand. The Rb^+^ cation is 10-coordinated by O atoms from eight 3,5-dinitro­salicylate anions and is linked by three μ_2_-O atoms, forming a zigzag chain along the *b*-axis direction, which is further linked by the phenyl groups, giving the three-dimensional framework. The crystal structure involves intra-anionic O—H⋯O hydrogen bonds and strong π–π stacking inter­actions [centroid-centroid distance = 3.6755 (7) Å].

## Related literature

For 3,5-dinitro­salicylate complexes, see: Hu *et al.* (2005 ▶); Song *et al.* (2007 ▶, 2008 ▶). For Rb–O bond lengths, see: Cametti *et al.* (2005 ▶). 
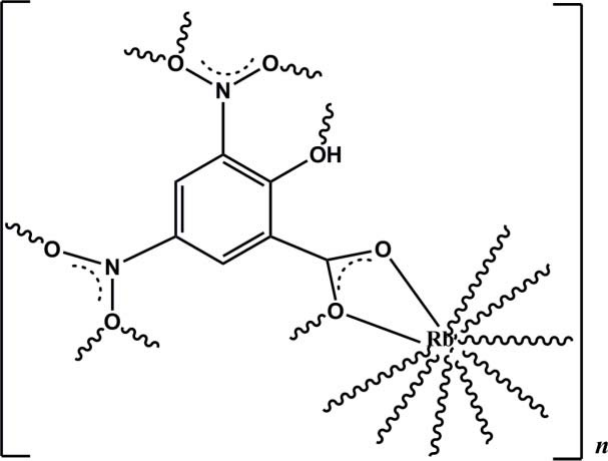


## Experimental

### 

#### Crystal data

[Rb(C_7_H_3_N_2_O_7_)]
*M* *_r_* = 312.58
Monoclinic, 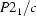

*a* = 7.5957 (15) Å
*b* = 7.2971 (15) Å
*c* = 17.036 (3) Å
β = 95.10 (3)°
*V* = 940.5 (3) Å^3^
*Z* = 4
Mo *K*α radiation
μ = 5.30 mm^−1^
*T* = 293 K
0.64 × 0.60 × 0.20 mm

#### Data collection

Bruker SMART CCD diffractometer
Absorption correction: multi-scan (*SADABS*; Sheldrick, 1996 ▶) *T*_min_ = 0.219, *T*_max_ = 0.548
8781 measured reflections
1715 independent reflections
1548 reflections with *I* > 2σ(*I*)
*R*_int_ = 0.065

#### Refinement

*R*[*F* ^2^ > 2σ(*F* ^2^)] = 0.044
*wR*(*F* ^2^) = 0.107
*S* = 1.07
1715 reflections
154 parameters
H-atom parameters constrained
Δρ_max_ = 0.59 e Å^−3^
Δρ_min_ = −1.28 e Å^−3^

### 

Data collection: *SMART* (Bruker, 2002 ▶); cell refinement: *SAINT* (Bruker, 2002 ▶); data reduction: *SAINT*; program(s) used to solve structure: *SHELXS97* (Sheldrick, 2008 ▶); program(s) used to refine structure: *SHELXL97* (Sheldrick, 2008 ▶); molecular graphics: *SHELXTL* (Sheldrick, 2008 ▶); software used to prepare material for publication: *SHELXTL*.

## Supplementary Material

Crystal structure: contains datablocks I, global. DOI: 10.1107/S1600536811008476/om2407sup1.cif

Structure factors: contains datablocks I. DOI: 10.1107/S1600536811008476/om2407Isup2.hkl

Additional supplementary materials:  crystallographic information; 3D view; checkCIF report

## Figures and Tables

**Table 1:** Hydrogen-bond geometry (Å, °)

*D*—H⋯*A*	*D*—H	H⋯*A*	*D*⋯*A*	*D*—H⋯*A*
O7—H7*A*⋯O2	0.85	1.67	2.459 (4)	153

**Fig. 1.**
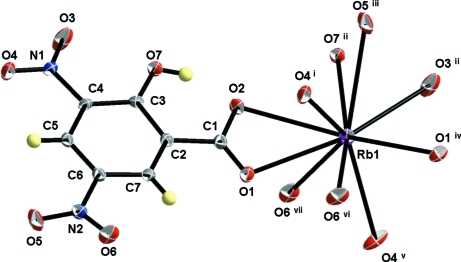
The structure of the title compound, showing the atomic numbering scheme. Non-H atoms are shown with 30% probability displacement ellipsoids. Symmetry code: (i) -x + 2, -y + 2, -z; (ii) -x + 3, -y + 2, -z; (iii) x + 1, y + 1, z; (iv) -x + 3, y + 1/2, -z + 1/2; (v) x + 1, -y + 3/2, z + 1/2; (vi) -x + 2, y + 1/2, -z + 1/2; (vii) x + 1, y, z.

**Fig. 2.**
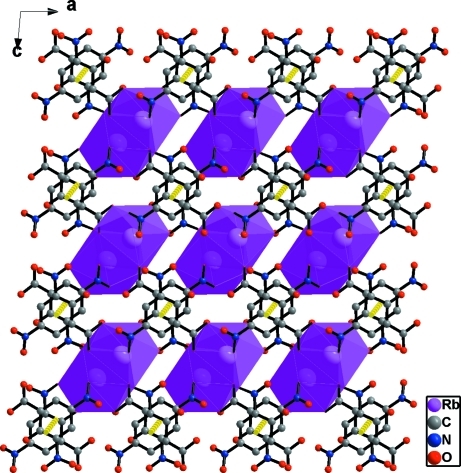
The three-dimensional framework of the title compound viewed along the b-axis direction, and the broken lines represent the π–π stacking interactions. The H atoms were omitted for clarity.

## supplementary crystallographic information

### Comment

In the structural investigation of 3,5-dinitrosalicylate complexes, it has been found that the 3,5-dinitrosalicylate moiety functions as a multidentate ligand (Hu *et al.*, 2005; Song *et al.*, 2007; Song *et al.*, 2008) with versatile bonding and coordination modes. In this paper, we report the crystal structure of the title compound, a new Rb complex obtained by the reaction of 3,5-dinitrosalicylic acid and RbOH in water.

The asymmetric unit of the title compound comprises a Rb cation, and a 3,5-dinitrosalicylate anion. The central cation is coordinated to ten O atoms from eight 3,5-dinitrosalicylate anions (Fig. 1) with the Rb–O distances ranging from 2.821 (3) Å to 3.385 (4) Å, which are well within the range reported in the literature (Cametti *et al.*, 2005). The Rb centre is firstly linked by three µ_2_-oygen atoms to form a one-dimensional zigzag-shaped chain along the *b*-axis direction, which is further linked by the phenyl groups to give the three-dimensional framework of the title compound (Fig. 2). The shortest intra-anionic hydrogen bond is established between O7–H7A···O2 with the bond distances of 2.459 (4) Å. Furthermore, strong aromatic π–π stacking interactions between adjacent phenyl rings with a center-to-center distance of 3.6755 (7) Å help to stabilize the three-dimensional framework.

### Experimental

Analysis grade 3,5-dinitrosalicylic acid and RbOH (purity > 99.5%, Sinopharm Chemical Reagent Co., Ltd., Shanghai, China) were commercially available and used without further purification. To a solution of 10 mmol 3,5-dinitrosalicylic acid in 50 ml double-distilled water, a solution of an equimolar amount of RbOH in 40 ml double-distilled water was added dropwise at room temperature. After vigorous stirring for 3 h, the resulting solution was evaporated to a volume of about 20 ml in vacuum and filtered hot. The filtrate was then set aside for crystallization at room temperature. One month later, yellow block crystals of the title compound suitable for X-ray determination were isolated.

### Refinement

Carbon-bound H atoms were placed at calculated positions and were treated as riding on the parent C atoms with C–H = 0.93 Å, and with *U*_iso_(H) = 1.2 *U*_eq_(C). Oxygen-bound H atoms were originally located in difference Fourier maps and were refined with distance restraints of O–H = 0.85 Å, and and with *U*_iso_(H) = 1.5 *U*_eq_(O).

### Crystal data

**Table:**

[Rb(C_7_H_3_N_2_O_7_)]	*F*(000) = 608
*M**_r_* = 312.58	*D*_x_ = 2.208 Mg m^−^^3^
Monoclinic, *P*2_1_/*c*	Mo *K*α radiation, λ = 0.71073 Å
Hall symbol: -P 2ybc	Cell parameters from 3447 reflections
*a* = 7.5957 (15) Å	θ = 3.0–25.4°
*b* = 7.2971 (15) Å	µ = 5.30 mm^−^^1^
*c* = 17.036 (3) Å	*T* = 293 K
β = 95.10 (3)°	Block, yellow
*V* = 940.5 (3) Å^3^	0.64 × 0.60 × 0.20 mm
*Z* = 4	

### Data collection

**Table:**

Bruker SMART CCD diffractometer	1715 independent reflections
Radiation source: fine-focus sealed tube	1548 reflections with *I* > 2σ(*I*)
graphite	*R*_int_ = 0.065
φ and ω scans	θ_max_ = 25.4°, θ_min_ = 3.0°
Absorption correction: multi-scan (*SADABS*; Sheldrick, 1996)	*h* = −9→9
*T*_min_ = 0.219, *T*_max_ = 0.548	*k* = −7→8
8781 measured reflections	*l* = −18→20

### Refinement

**Table:**

Refinement on *F*^2^	Primary atom site location: structure-invariant direct methods
Least-squares matrix: full	Secondary atom site location: difference Fourier map
*R*[*F*^2^ > 2σ(*F*^2^)] = 0.044	Hydrogen site location: inferred from neighbouring sites
*wR*(*F*^2^) = 0.107	H-atom parameters constrained
*S* = 1.07	*w* = 1/[σ^2^(*F*_o_^2^) + (0.0621*P*)^2^] where *P* = (*F*_o_^2^ + 2*F*_c_^2^)/3
1715 reflections	(Δ/σ)_max_ = 0.001
154 parameters	Δρ_max_ = 0.59 e Å^−^^3^
0 restraints	Δρ_min_ = −1.28 e Å^−^^3^

### Special details

**Table:**

Geometry. All e.s.d.'s (except the e.s.d. in the dihedral angle between two l.s. planes) are estimated using the full covariance matrix. The cell e.s.d.'s are taken into account individually in the estimation of e.s.d.'s in distances, angles and torsion angles; correlations between e.s.d.'s in cell parameters are only used when they are defined by crystal symmetry. An approximate (isotropic) treatment of cell e.s.d.'s is used for estimating e.s.d.'s involving l.s. planes.
Refinement. Refinement of *F*^2^ against ALL reflections. The weighted *R*-factor *wR* and goodness of fit *S* are based on *F*^2^, conventional *R*-factors *R* are based on *F*, with *F* set to zero for negative *F*^2^. The threshold expression of *F*^2^ > σ(*F*^2^) is used only for calculating *R*-factors(gt) *etc*. and is not relevant to the choice of reflections for refinement. *R*-factors based on *F*^2^ are statistically about twice as large as those based on *F*, and *R*- factors based on ALL data will be even larger.

### Fractional atomic coordinates and isotropic or equivalent isotropic displacement parameters (Å^2^)

**Table:**

	*x*	*y*	*z*	*U*_iso_*/*U*_eq_	
Rb1	1.61252 (5)	1.14921 (5)	0.20167 (2)	0.0369 (2)	
C1	1.2759 (5)	0.8926 (5)	0.1057 (2)	0.0273 (8)	
C2	1.1105 (5)	0.8137 (5)	0.0645 (2)	0.0242 (8)	
C3	1.0990 (5)	0.7877 (5)	−0.0199 (2)	0.0241 (8)	
C4	0.9370 (5)	0.7063 (5)	−0.0527 (2)	0.0247 (8)	
C5	0.8025 (5)	0.6593 (4)	−0.0081 (2)	0.0251 (8)	
H5	0.6998	0.6056	−0.0314	0.030*	
C6	0.8215 (5)	0.6929 (5)	0.0721 (2)	0.0248 (8)	
C7	0.9735 (5)	0.7708 (5)	0.1085 (2)	0.0240 (8)	
H7	0.9826	0.7939	0.1624	0.029*	
N1	0.9095 (5)	0.6663 (4)	−0.13724 (19)	0.0310 (8)	
N2	0.6787 (5)	0.6448 (4)	0.1190 (2)	0.0335 (8)	
O1	1.2938 (4)	0.9117 (4)	0.17699 (15)	0.0361 (7)	
O2	1.3980 (3)	0.9418 (4)	0.06105 (15)	0.0377 (7)	
O3	1.0271 (5)	0.6878 (6)	−0.17888 (19)	0.0680 (11)	
O4	0.7641 (5)	0.6080 (5)	−0.16227 (18)	0.0562 (9)	
O5	0.5666 (4)	0.5376 (5)	0.09310 (19)	0.0554 (9)	
O6	0.6775 (4)	0.7154 (5)	0.18481 (17)	0.0500 (8)	
O7	1.2245 (4)	0.8369 (4)	−0.05933 (16)	0.0356 (7)	
H7A	1.3073	0.8813	−0.0282	0.053*	

### Atomic displacement parameters (Å^2^)

**Table:**

	*U*^11^	*U*^22^	*U*^33^	*U*^12^	*U*^13^	*U*^23^
Rb1	0.0346 (3)	0.0485 (3)	0.0288 (3)	−0.01148 (17)	0.0097 (2)	−0.00679 (15)
C1	0.022 (2)	0.0290 (18)	0.031 (2)	−0.0011 (16)	0.0012 (16)	0.0035 (15)
C2	0.023 (2)	0.0228 (17)	0.027 (2)	−0.0013 (15)	0.0019 (16)	0.0008 (14)
C3	0.025 (2)	0.0243 (17)	0.0238 (18)	0.0021 (16)	0.0059 (15)	0.0050 (15)
C4	0.031 (2)	0.0272 (18)	0.0160 (17)	0.0016 (16)	0.0020 (15)	0.0024 (14)
C5	0.020 (2)	0.0239 (18)	0.031 (2)	−0.0034 (14)	−0.0018 (16)	0.0013 (14)
C6	0.021 (2)	0.0265 (17)	0.028 (2)	−0.0020 (15)	0.0036 (15)	0.0017 (15)
C7	0.027 (2)	0.0227 (17)	0.0233 (17)	−0.0006 (15)	0.0049 (15)	0.0017 (14)
N1	0.032 (2)	0.0367 (19)	0.0237 (17)	−0.0022 (14)	0.0004 (16)	0.0025 (13)
N2	0.026 (2)	0.042 (2)	0.033 (2)	−0.0057 (15)	0.0073 (16)	0.0027 (14)
O1	0.0316 (16)	0.0488 (17)	0.0275 (15)	−0.0104 (14)	0.0005 (12)	−0.0017 (12)
O2	0.0226 (14)	0.0557 (18)	0.0351 (15)	−0.0144 (14)	0.0048 (11)	0.0052 (13)
O3	0.042 (2)	0.136 (4)	0.0265 (17)	−0.015 (2)	0.0110 (16)	−0.0129 (18)
O4	0.054 (2)	0.086 (2)	0.0274 (16)	−0.0344 (19)	−0.0043 (15)	−0.0013 (15)
O5	0.0378 (18)	0.070 (2)	0.060 (2)	−0.0309 (17)	0.0149 (15)	−0.0087 (17)
O6	0.0384 (19)	0.086 (2)	0.0284 (16)	−0.0061 (17)	0.0174 (13)	−0.0040 (16)
O7	0.0283 (17)	0.0526 (18)	0.0267 (15)	−0.0122 (12)	0.0067 (12)	0.0018 (11)

### Geometric parameters (Å, °)

**Table:**

Rb1—O7^i^	2.821 (3)	C5—C6	1.383 (5)
Rb1—O1^ii^	2.861 (3)	C5—H5	0.9300
Rb1—O1	2.977 (3)	C6—C7	1.383 (5)
Rb1—O3^i^	3.041 (4)	C6—N2	1.447 (5)
Rb1—O6^iii^	3.096 (3)	C7—H7	0.9300
Rb1—O4^iv^	3.122 (3)	N1—O3	1.199 (5)
Rb1—O2	3.161 (3)	N1—O4	1.224 (4)
Rb1—O6^v^	3.221 (4)	N2—O5	1.210 (4)
Rb1—O4^vi^	3.381 (4)	N2—O6	1.234 (4)
Rb1—O5^vii^	3.385 (4)	O1—Rb1^viii^	2.861 (3)
C1—O1	1.218 (4)	O3—Rb1^i^	3.041 (4)
C1—O2	1.301 (5)	O4—Rb1^ix^	3.122 (3)
C1—C2	1.498 (5)	O4—Rb1^vi^	3.381 (4)
C2—C7	1.371 (5)	O5—Rb1^x^	3.385 (4)
C2—C3	1.446 (5)	O6—Rb1^xi^	3.096 (3)
C3—O7	1.266 (5)	O6—Rb1^xii^	3.221 (4)
C3—C4	1.434 (5)	O7—Rb1^i^	2.821 (3)
C4—C5	1.370 (5)	O7—H7A	0.8500
C4—N1	1.465 (5)		
			
O7^i^—Rb1—O1^ii^	119.80 (8)	O4^vi^—Rb1—O5^vii^	53.74 (8)
O7^i^—Rb1—O1	108.25 (8)	O1—C1—O2	122.0 (3)
O1^ii^—Rb1—O1	129.70 (4)	O1—C1—C2	121.7 (3)
O7^i^—Rb1—O3^i^	53.65 (9)	O2—C1—C2	116.4 (3)
O1^ii^—Rb1—O3^i^	70.22 (9)	C7—C2—C3	122.1 (3)
O1—Rb1—O3^i^	160.00 (9)	C7—C2—C1	118.5 (3)
O7^i^—Rb1—O6^iii^	157.29 (9)	C3—C2—C1	119.4 (3)
O1^ii^—Rb1—O6^iii^	65.76 (9)	O7—C3—C4	124.8 (3)
O1—Rb1—O6^iii^	64.16 (8)	O7—C3—C2	120.6 (3)
O3^i^—Rb1—O6^iii^	135.84 (10)	C4—C3—C2	114.6 (3)
O7^i^—Rb1—O4^iv^	119.92 (9)	C5—C4—C3	122.9 (3)
O1^ii^—Rb1—O4^iv^	79.29 (9)	C5—C4—N1	116.5 (3)
O1—Rb1—O4^iv^	89.80 (9)	C3—C4—N1	120.6 (3)
O3^i^—Rb1—O4^iv^	93.04 (11)	C4—C5—C6	119.1 (3)
O6^iii^—Rb1—O4^iv^	82.29 (9)	C4—C5—H5	120.4
O7^i^—Rb1—O2	66.53 (7)	C6—C5—H5	120.4
O1^ii^—Rb1—O2	161.17 (8)	C5—C6—C7	121.7 (4)
O1—Rb1—O2	41.94 (7)	C5—C6—N2	119.0 (3)
O3^i^—Rb1—O2	119.98 (8)	C7—C6—N2	119.2 (3)
O6^iii^—Rb1—O2	101.58 (8)	C2—C7—C6	119.5 (3)
O4^iv^—Rb1—O2	113.97 (9)	C2—C7—H7	120.3
O7^i^—Rb1—O6^v^	82.90 (7)	C6—C7—H7	120.3
O1^ii^—Rb1—O6^v^	133.80 (8)	O3—N1—O4	122.4 (3)
O1—Rb1—O6^v^	62.88 (8)	O3—N1—C4	120.4 (3)
O3^i^—Rb1—O6^v^	103.08 (10)	O4—N1—C4	117.2 (3)
O6^iii^—Rb1—O6^v^	109.40 (9)	O5—N2—O6	122.6 (3)
O4^iv^—Rb1—O6^v^	54.97 (8)	O5—N2—C6	119.5 (3)
O2—Rb1—O6^v^	62.26 (8)	O6—N2—C6	117.9 (3)
O7^i^—Rb1—O4^vi^	103.81 (8)	C1—O1—Rb1^viii^	130.0 (3)
O1^ii^—Rb1—O4^vi^	86.82 (8)	C1—O1—Rb1	103.0 (2)
O1—Rb1—O4^vi^	67.29 (9)	Rb1^viii^—O1—Rb1	98.09 (8)
O3^i^—Rb1—O4^vi^	121.68 (10)	C1—O2—Rb1	92.0 (2)
O6^iii^—Rb1—O4^vi^	53.55 (8)	N1—O3—Rb1^i^	148.5 (3)
O4^iv^—Rb1—O4^vi^	135.41 (5)	N1—O4—Rb1^ix^	136.8 (3)
O2—Rb1—O4^vi^	74.36 (8)	N1—O4—Rb1^vi^	127.5 (3)
O6^v^—Rb1—O4^vi^	129.12 (8)	Rb1^ix^—O4—Rb1^vi^	85.29 (8)
O7^i^—Rb1—O5^vii^	62.09 (8)	N2—O5—Rb1^x^	107.8 (3)
O1^ii^—Rb1—O5^vii^	80.87 (8)	N2—O6—Rb1^xi^	124.4 (2)
O1—Rb1—O5^vii^	111.55 (8)	N2—O6—Rb1^xii^	120.3 (2)
O3^i^—Rb1—O5^vii^	69.70 (10)	Rb1^xi^—O6—Rb1^xii^	88.53 (8)
O6^iii^—Rb1—O5^vii^	99.57 (8)	C3—O7—Rb1^i^	151.1 (2)
O4^iv^—Rb1—O5^vii^	157.25 (9)	C3—O7—H7A	109.0
O2—Rb1—O5^vii^	88.03 (8)	Rb1^i^—O7—H7A	99.6
O6^v^—Rb1—O5^vii^	141.57 (7)		

Symmetry codes: (i) −*x*+3, −*y*+2, −*z*; (ii) −*x*+3, *y*+1/2, −*z*+1/2; (iii) −*x*+2, *y*+1/2, −*z*+1/2; (iv) *x*+1, −*y*+3/2, *z*+1/2; (v) *x*+1, *y*, *z*; (vi) −*x*+2, −*y*+2, −*z*; (vii) *x*+1, *y*+1, *z*; (viii) −*x*+3, *y*−1/2, −*z*+1/2; (ix) *x*−1, −*y*+3/2, *z*−1/2; (x) *x*−1, *y*−1, *z*; (xi) −*x*+2, *y*−1/2, −*z*+1/2; (xii) *x*−1, *y*, *z*.

### Hydrogen-bond geometry (Å, °)

**Table:**

*D*—H···*A*	*D*—H	H···*A*	*D*···*A*	*D*—H···*A*
O7—H7A···O2	0.85	1.67	2.459 (4)	153
